# Restraint use among selected hospitalized elderly patients in Cairo, Egypt

**DOI:** 10.1186/s13104-017-2978-x

**Published:** 2017-11-28

**Authors:** Amira G. Eltaliawi, Mohamed El-Shinawi, Angela Comer, Sarah Hamazah, Jon Mark Hirshon

**Affiliations:** 1 Palestine Hospital, Cairo, Egypt; 2 General Surgery Department, Ain Shams University, Cairo, Egypt; 3 Charles McC. Mathias Jr., National Study Center for Trauma and Emergency Medical Systems, University of Maryland School of Medicine, Baltimore, MD USA; 4 Department of Geriatrics, Ain Shams University, Cairo, Egypt; 5 Department of Emergency Medicine, University of Maryland School of Medicine, Baltimore, MD USA

**Keywords:** Restraints, Elderly, Caregiver, Egypt

## Abstract

**Objective:**

This study’s primary objective was to investigate the prevalence of physical and chemical restraint use in selected elderly hospitalized patients.

**Results:**

This study was conducted in April 2014 in four major acute care hospitals. Trained data collectors assessed the use of physical and chemical restraint among all admitted elderly patients. There were 287 elderly patients (median age 64 years, 46% women). 32 patients were restrained. The overall prevalence of restraints was 11.1%, with physical restraint use alone at 3.2% and chemical restraints use alone at 7.3%. Restraint use varied by hospital type, with the highest at the private hospital (22.9%) and the lowest at the two university hospitals (< 6%). In conclusion the prevalence of physical and chemical restraint use among admitted elderly patients in Egypt is comparable to that seen in developed countries. However, the use appears to vary widely by hospital type. The use of restraints in the elderly remains an important question considering the increasing number of elderly.

**Electronic supplementary material:**

The online version of this article (10.1186/s13104-017-2978-x) contains supplementary material, which is available to authorized users.

## Introduction

Physical and chemical restraints are commonly used in acute care hospitals mostly to prevent falls or patients’ interference with medical care, despite the association of restraints with adverse consequences. Evidence demonstrates that the use of physical restraints can lead to multiple negative effects as pressure sores, decline in functional and cognitive state [1] as well as negative emotional effects on patients and their families [2]. The elderly are a particularly vulnerable population due to their decreased functional reserve [3]. Chemical restraints can have negative consequences as delirium and falls [4], in addition to potential risk of increased mortality [5].

Despite their risks, restraints are commonly used in acute care hospitals. Among the patient-related factors linked to restraint use are age, gender, functional dependency, medical diagnoses, aggression and self-harm [6]. Nurse-related factors include perceived fall risk [7] and high nurse-to-patient ratios [8]. Side rails, tables and belts are the most common used physical restraints [9]. While these factors are well documented in western settings, they have yet to be adequately investigated in the Middle East.

Physical restraints are considered an acceptable practice in many settings [10].The reported prevalence worldwide is highest in intensive care units (9–39%) and nursing homes (41–64%) [9]. In comparison, the prevalence of chemical restraints in long-term care facilities in the U.S. is slightly lower with an upper range of 34% [11].Egypt as the most populous Arab country with a growing elderly population, and one of the few Middle Eastern countries where organized geriatric care is available, presents a compelling opportunity to study this topic as data do not currently exist.

## Main text

### Methods

#### Study design

We conducted an observational cross-sectional study at four large acute care hospitals in Egypt to assess the prevalence of physical and chemical restraint use in elderly patients. Institutional Review Board approval was obtained from Ain Shams University in Cairo, Egypt and University of Maryland, Baltimore in Maryland, United States. The Ain Shams approval was accepted for data collection at all sites within Cairo. Verbal consent was felt appropriate, as the data collected were (1) anonymous, (2) observational and (3) retrospective. Consent for medical record review was waived, as it was a retrospective, observation, de-identified study. Verbal consent was obtained from the caregiver of the patients, when present, as the caregiver was also a research participant as well as from the nurses present on the ward. These consent processes were deemed appropriate considering that the only potential document linking the participants to the research would have been the consent form, potentially leading to increased risk of participant identification.

#### Setting and study population

A convenience sample of four acute care hospitals participated in the study in April 2014: 2 university hospitals, a ministerial (Ministry of Health) public hospital, and a private hospital. These hospitals represent three major acute care hospital types that exist in Cairo: university, public and private. The study population consisted of all elderly patients (age > 55) admitted to all wards at the time of data collection, however patients in psychiatric wards, intensive care units, and emergency departments were excluded from this study.

#### Definition of physical and chemical restraints

Physical restraints were defined as “any device, material or equipment attached to or near a person’s body and which cannot be controlled or easily removed by the person and which deliberately prevents or is deliberately intended to prevent a person’s free body movement to a position of choice and/or a person’s normal access to their body [11].

Chemical restraints in this study were limited to patient’s given sedative hypnotics (benzodiazepines, non-benzodiazepines, and barbiturates), typical and atypical antipsychotics [12].

#### Data collection

Data collectors were trained on what constituted a physical or chemical restraint and how to conduct interviews. A close-ended questionnaire was developed for this study by researchers specializing in geriatric medicine, adapted from questionnaires used in previous studies [13, 14]. A pilot study to validate the questionnaire was then conducted on a single ward. During the pilot study, it was observed that most patients had caregivers with them; the caregivers were sometimes assigned by the nurses to help in observing patients. Thus, a caregiver interview was included in the final study in an attempt to further understand this relation and its effects. The results of this pilot study were not included in the study.

Data collectors visited the study hospitals on a single, specific midweek day, each hospital on separate day, overlapping afternoon and evening shifts (between 4 p.m. and 10 p.m.) in order to assess conditions when nurse/patient ratios were at its lowest. The staffs were not aware when DCs were to come and collect data. DCs observed patients, interviewed caregivers (both informal as family members and formal as paid caretakers), interviewed nurses and reviewed the medical charts. DCs visited patients to determine physical restraint use. Caregivers’ presence was documented; they were asked if nurses required their presence. Nurses were asked about type of and reason for physical restraint use. Reasons for their use were listed according to five most important reasons found in literature, i.e.: (1) confusion, (2) prevention of falls, (3) prevention of wandering, (4) behavior management/prevention of disruption and (5) an “others” category [14]. The use of chemical restraint was determined from chart and reasons were determined through nurse interview. The Charlson Comorbidity index was used to assess comorbidities and required elements were extracted from the chart and then scored [15].

#### Data analysis

Univariate analyses were conducted to examine the distribution of data elements by hospital. Age, number of medications, and Charlson Comorbidity Index were not normally distributed by hospital, so medians and interquartile ranges (IQR) were reported. Variables for each hospital were compared to the private hospital using Chi square and Wilcoxon tests for significance.

Bivariate analysis comparing the demographic and clinical data between the restraint group and the non-restraint group was done using the non-parametric Wilcoxon signed-rank test for continues variables and by Chi square test for categorical data. Two by two tables were constructed to investigate the significance of caregiver presence in the restrained and non-restrained groups with Chi square and Fischer exact to test for significance. Statistical analysis was performed using SAS statistical software (Version 9.2), SAS Institute Inc., Cary, NC, USA. Significance was set at a P < 0.05.

### Results

There were 287 elderly patients [median age 64 years (IQR: 60–70), 46% women] identified for study inclusion (Table 1). Median age was greater in the private and ministerial hospital than in the university hospitals. The number of medications in general was highest at the ministerial hospital (9.3 medications/patient) and lowest at university hospital B. The highest percentage of informal caregivers was at university hospital A (80%) and the lowest was at the private hospital (66%).

**Table 1:** Patient characteristics during midweek ward visits from selected hospitals, Cairo, Egypt, April 2014

	University Hospital A (N = 113)	University Hospital B (N = 69)	Ministerial Hospital (N = 57)	Private Hospital (N = 48)
Median age (IQR)	60 (58–67)*	63 (58–68)*	67 (62–73)	68.5 (63–75.5)
Male sex (N%)	51 (45.1)	45 (65.2)	29 (50.9)	30 (62.5)
Education (N%)				
Illiterate	75 (66.4)*	39 (56.5)*	28 (49.1)*	9 (18.8)
Some school	29 (25.7)	21 (30.4)	21 (36.8)	19 (39.6)
College	8 (7.1)	9 (13.0)	6 (10.5)	14 (29.2)
Post graduate	1 (0.9)	0	2 (3.5)	4 (8.3)
Age adjusted Charlson comorbidities score				
Median score (IQR)	6.0 (5–8)	5.5 (4–7)*	6.0 (5–7)*	7.0 (6–9)
Scores by categories				
< 5 (N%)	17 (15.0)	23 (33.8)*	9 (15.8)*	3 (6.3)
5–6 (N%)	27 (23.9)	11 (16.2)	8 (14.0)	8 (16.7)
6–8 (N%)	28 (24.8)	23 (33.8)	29 (50.9)	16 (33.3)
> 8 (N%)	41 (36.3)	11 (16.2)	11 (19.3)	21 (43.8)
Device (N%)				
None	24 (21.2)*	10 (14.5)	30 (52.6)*	5 (10.4)
Feeding tube	69 (61.1)	21 (30.4)	17 (29.8)	16 (33.3)
Urinary catheter	17 (15.0)	32 (46.4)	8 (14.0)	21 (43.8)
Tracheal tube	3 (2.7)	6 (8.7)	2 (3.5)	6 (12.5)
Medications				
Median number per patient (IQR)	7.0 (5–10)	4.0 (3–6)*	8.0 (7–11)	8.0 (4–12)
Attempts at interference in treatment (N%)				
More than once	3 (2.7)	1 (1.5)	0 (0)	0 (0)
Once	7 (6.2)	6 (8.7)	2 (3.5)	4 (8.3)
None	103 (91.2)	62 (89.9)	55 (96.5)	44 (91.7)
Successful attempts at interference (N%)				
0	106 (93.8)	63 (91.3)	55 (96.5)	44 (91.7)
1	6 (5.3)	4 (5.8)	1 (1.8)	4 (8.3)
2	0	0	1 (1.8)	0
4	1 (0.9)	0	0	0
5	0	1 (1.5)	0	0
20	0	1 (1.5)	0	0
Number of falls				
None	106 (94.6)	67 (97.1)	54 (94.7)	48 (100)
Once	4 (3.6)	2 (2.9)	3 (5.3)	0
More than once	2 (1.8)	0	0	0
Caregiver present (N%)				
Formal	1 (0.9)	1 (1.5)	1 (1.8)	0
Informal	91 (80.5)	52 (75.4)	36 (63.2)	32 (66.7)
None	21 (18.6)	16 (23.2)	20 (35.1)	16 (33.3)
Caregiver asked to observe patient (N%)	35 (31.0)*	35 (55.7)	30 (52.6)	26 (54.2)

Specific variable totals may not equal column total due to missing data

* Statistically significant (p < 0.05) difference compared to the private hospital, which was used as the comparator

The hospitals with the highest percentage of elderly patients were the private hospital (54.5% of ward patients), and the university hospital A (45.4% of ward patients). Both hospitals consisted primarily of medical wards. The highest nurse-to-patient ratio was at the private hospital with one nurse for every six patients. The lowest was at university hospital B with one nurse for every 20 patients (Table 2).

**Table 2:** Facility characteristics during midweek visits to participating hospitals, Cairo, Egypt, April 2014

	University Hospital A	University Hospital B	Ministerial Hospital	Private Hospital	Total
Nurse to patient ratio (%)	0.08	0.05	0.06	0.16	
Number of patients (N)	249	298	251	88	886
Number of elderly patients (N)	113	69	57	48	287

Thirty-two patients were restrained (9 physically, 21 chemically, 2 both chemical and physical). The overall prevalence of any restraint use was 11.1%. The overall prevalence of physical restraint alone was 3.2%, which was highest at the ministerial hospital (8.8%) and lowest at both university hospital A (0%) and the private hospital (0%). The overall prevalence of chemical restraint alone was 7.3% and prevalence of both physical and chemical restraint was 0.7% and only occurred at the private hospital (Table 3).

**Table 3:** Prevalence of physical and chemical restraint use in four different acute hospitals during midweek visits, Cairo, Egypt, April 2014

	Physical restraint use N (%)	Chemical restraint use N (%)	Both physical and chemical restraint use N (%)	Any restraint use N (%)
University Hospital A (N = 113)	0 (0)	6 (5.3)	0 (0)	6 (5.3)
University Hospital B (N = 69)	4 (5.8)	0 (0)	0 (0)	4 (5.8)
Ministerial Hospital (N = 57)	5 (8.8)	6 (10.5)	0 (0)	11 (19.3)
Private Hospital (N = 48)	0 (0)	9 (18.8)	2 (4.1)	11 (22.9)
Total	9 (3.1)	21 (7.3)	2 (0.7)	32 (11.1)

Bivariate analysis indicated that mean age of 55–64 was the only statistically significant factor observed for physical restraint use (Additional file 1: Table S1). Concerning chemical restraint use, a Charlson Index score of 7.0 and median number of medications of 13 (IQR: 7–16) proved to be statistically significant. The most cited reasons for use were prevention of falls and prevention of disruption of treatment (data not presented). The bivariate analysis of restraint use (both physical and chemical) did not find any statistical significance for presence or absence of a caregiver (Additional file 1: Table S2).

### Discussion

We found that the use of any type of restraint ranged from 5.3 to 22.9% per hospital with an average use across the four hospitals of 11.1%. The highest rate of any type of restraint usage was at the private hospital (22.9%). The overall prevalence rate for just physical restraints use was 3.2%, which varied from 0.0 to 8.8%, while the overall use of just chemical restraints was 7.3%, which varied from 5.3 to 18.8%.

The overall range for the use of restraints is lower than that reported in a review by Hammers et al. in 2005 in acute care settings; which reported a prevalence of physical restraint use between 33 and 68%. However, the prevalence of physical restraint use in Egypt as reported in this study is comparable to prevalence rates from similar studies done in other countries, e.g., 5% in the United States [14], 4% in Europe [16], despite higher patient to nurse ratios, less financial resources, and an absence of formal governmental regulations concerning restraint use. In a multi-center study conducted in acute care hospitals in Germany, the prevalence rate for physical restraint use was 11.8%, which is also comparable to the prevalence rates found in our study. However, in our study, bed rails were included, which could explain the overall higher prevalence rate of restraint use [17].

Prevalence of physical restraint use was highest in the ministerial hospital (8.8%). This may be due, to the more limited resources in public hospitals and the fact that these hospitals are referred complicated surgical cases. The lowest use was at the private hospital (0%, physical only; 4.1% combined physical and chemical) where the patient-to-nurse ratio was the lowest. However, the use of chemical restraints was also much higher in private hospitals (18.8%, chemical only) leading to an overall restraint use of 22.9%.

For physical restraints use, the only statistically significant patient characteristic was the age group 55–64. This could be due in part to the low median age among hospitalized elderly in Egypt. The presence of a caregiver, however, did not prove to be statistically significant in our study which may correlate with the results of a study conducted in 13 German nursing homes, where the attitudes of family members of residents were assessed towards physical restraint and were found to be more positive towards the use of physical restraints compared to nurses’ attitudes [18].

The total prevalence of chemical restraint use was 7.3%. It was highest at the private hospital (18.8%), which may reflect their greater availability. It was lowest at university hospital B (0%), possible due to the lack of availability of chemical restraints. In the bivariate analysis, chemical restraints showed a statistically significant relation with mean number of medications, which may reflect the effect of polypharmacy. These results can be correlated with a retrospective study in medical teaching units in Canada, which puts the incidence rate of chemical restraints at 10.3% [19]. Fear of falls and disruption of treatment were the most common indications for chemical restraints, which are consistent with the main reasons for restraint use in a study conducted to assess effect of education on use of restraints which were “protecting patients from falling of beds and chairs” [20].

### Conclusions

This study is the first to document the prevalence rates of physical and chemical restraint use among admitted elderly patients in Egypt and one of a limited number published in the Middle East. The use of physical and chemical restraints in elderly hospitalized patients in Egypt is similar to that found in developed countries. Factors found associated with restraint use included age, increased Charlson Morbidity Score and a greater number of medications used. Presence of a caregiver was not associated with use of restraints. Additional research should confirm these findings and explore additional factors related to restraint use. The use of restraints remains an important question considering the increasing number of elderly in Egypt and globally.

#### Limitations

The main limitation is that it is a convenience sample of a small number of hospitals in Cairo. Additionally, the afternoon visits on a single day may or may not have sampled a typical day. Despite the diversity of hospital settings, these limitations may introduce a selection bias when generalizing these findings to other hospitals and hospital systems in Cairo specifically and in Egypt generally. Additionally, Egypt as a single country may not fully represent practices in the Middle East. This may impact generalizability to other countries. However, despite these limitations, these findings should be considered as Egyptian hospitals and hospital systems develop policies related to the use of physical and chemical restraints, particularly for the elderly.

## Additional file

**Additional file 1: Table S1.** Physical or chemical restraint use as compared to patient characteristics from selected hospitals, Cairo, Egypt in April 2014. **Table S2.** Physical or chemical restraint use compared to whether or not caregiver was present, Cairo, Egypt in April 2014.

## Abbreviations

DCs: data collectors
IQR: interquartile ranges
SAS: Statistical Analysis Software

## References

[1] Evans D, Wood J, Lambert L (2002). A review of physical restraint minimization in the acute and residential care settings. J Adv Nurs.

[2] Bray K, Hill K, Robson W, Leaver G, Walker N, O’Leary M, Delaney T, Walsh D, Gager M, Waterhouse C, British Association of Critical Care Nurses (2004). British Association of Critical Care Nurses position statement on the use of restraint in adult critical care units. Nurs Crit Care.

[3] Gastmans C, Milisen K (2006). Use of physical restraint in nursing homes: clinical-ethical considerations. J Med Ethics.

[4] Kane RL, Ouslander JG, Abrass IB, Resnick B. Chapter 14. Drug therapy. In: editors. Essentials of Clinical Geriatrics, 7e. New York: McGraw-Hill; 2013. http://accessmedicine.mhmedical.com.proxy-hs.researchport.umd.edu/content.aspx?bookid=678&sectionid=44833893.

[5] FDA Public Health Advisory. Deaths with antipsychotics in elderly patients with behavioral disturbances. https://www.fda.gov/drugs/drugsafety/postmarketdrugsafetyinformationforpatientsandproviders/ucm053171.

[6] Bredthauser D, Becker C, Eichner B, Koczy P & Nikolaus T. Factors relating to the use of physical restraints in psychogeriatric care: a paradigm for elder abuse. Zeitschrift fur Gerontologie and Geriatrie. 2005; 38(2): 10–18. Erratum in: Z Gerontol Geriatr. 2005; 38(2): 151. 10.1007/s00391-005-0285-y 15756482

[7] Capezuti E, Strumpf NE, Evans LK, Grisso JA, Maislin G (1998). The relationship between physical restraint removal and falls and injuries among nursing home residents. J Gerontol A Biol Sci Med Sci.

[8] Demir A (2007). Nurses’ use of physical restraints in four Turkish hospitals. J Nurs Scholarsh.

[9] Hamers JP, Huizing AR (2005). Why do we use physical restraints in the elderly?. Z Gerontol Geriatr..

[10] Martin B, Mathisen L (2005). Use of physical restraints in adult critical care: a bicultural study. Am J Crit Care.

[11] Hughes CM, Lapane KL (2005). Administrative initiatives for reducing inappropriate prescribing of psychotropic drugs in nursing homes: how successful have they been?. Drugs Aging.

[12] Phillips CD, Spry KM, Sloane PD, Hawes C (2000). Use of physical restraints and psychotropic medications in Alzheimer special care units in nursing homes. Am J Public Health.

[13] Chiba Y, Yamamoto-Mitani N, Kawasaki M (2012). A national survey of the use of physical restraint in long-term care hospitals in Japan. J Clin Nurs.

[14] Minnick AF, Mion LC, Leipzig R, Lamb K, Palmer RM (1998). Prevalence and patterns of physical restraint use in the acute care setting. J Nurs Adm.

[15] Charlson M, Szatrowski TP, Peterson J, Gold J (1994). Validation of a combined comorbidity index. J Clin Epidemiol.

[16] de Vries OJ, Ligthart GJ, European Academy of Medicine of Ageing-Course III (2004). Nikolaus. Differences in period prevalence of the use of physical restraints in elderly inpatients of European hospitals and nursing homes. J Gerontol Series A Biol Sci Med Sci..

[17] Kruger C, Mayer H, Haastert B, Meyer G (2013). Use of physical restraints in acute hospitals in Germany: a multicentre cross-sectional study. Int J Nurs Stud.

[18] Haut A, Kolbe N, Strupeit S, Mayer H, Meyer G (2010). Attitudes of relatives of nursing home residents toward physical restraints. J Nurs Scholarsh.

[19] Kow JV, Hogan DB (2000). Use of physical and chemical restraints in medical teaching units. CMAJ.

[20] Johnson Kari, Curry Valerie, Steubing Alison, Diana S, McCray A, McFarren A, Domb A (2015). A non-pharmacologic approach to decrease restraint use. Intensive Crit Care Nurs.

