# Supplementary material for: Restraint use among selected hospitalized elderly patients in Cairo, Egypt
Source: BMC Res Notes. 2017 Nov 28;10:633. doi: 10.1186/s13104-017-2978-x (PMC5704368; doi:10.1186/s13104-017-2978-x)
Supplement: Supplementary file 1 — Additional file 1: Table S1. Physical or chemical restraint use as compared to patient characteristics from selected hospitals, Cairo, Egypt in April 2014. Table S2. Physical or chemical restraint use compared to whether or not caregiver was present, Cairo, Egypt in April 2014. [file 13104_2017_2978_MOESM1_ESM.docx]

**Table S1: Physical or chemical restraint use as compared to patient characteristics from selected hospitals, Cairo, Egypt in April 2014.**

|  |  | **Physical Restraints (excluding bedrails)**  **N=9** | **Chemical Restraints**  **N=21** | **None**  **N=257** |
| --- | --- | --- | --- | --- |
| **Age** | 55-64 | 4 (44.4)* | 8 (38.1) | 133 (52.0) |
|  | 65-74 | 2 (22.2) | 7 (33.3) | 91 (35.6) |
|  | 75-84 | 1 (11.1) | 6 (28.6) | 28 (10.9) |
|  | 85+ | 2 (22.2) | 0 (0) | 4 (1.6) |
| **Sex** | Male | 2 (22.2) | 13 (61.9) | 140 (54.5) |
|  | Female | 7 (77.8) | 8 (38.1) | 113 (44.0) |
| **Charlson Index** |  | 6.0 (6-8) | 7.0 (6-8)* | 6.0 (5-8.) |
| **Devices** | None | 4 (44.4) | 1 (4.8)* | 64 (24.9) |
|  | Feeding Tube | 1 (11.1) | 10 (47.6) | 112 (43.6) |
|  | Urinary Catheter | 4 (44.4) | 10 (47.6) | 64 (24.9) |
|  | Tracheal Tube | 0 | 0 | 17 (6.6) |
| **Median No. of Medications (IQR)** |  | 8.0 (5-10) | 13 (7-16)* | 7.0(4-9) |

* Statistically significant (p<0.05) difference compared to None, which was used as the comparator.

**^+^**Specific variable totals may not equal column total due to missing data.

**Table S2: Physical or chemical restraint use compared to whether or not caregiver was present, Cairo, Egypt in April 2014.**

| **Χ^2^ p-value** | **Chemical Restraint** | | **Fisher’s exact p-value** | **Physical Restraint** | |  |
| --- | --- | --- | --- | --- | --- | --- |
|  | Not restrained | Restrained |  | Not restrained | Restrained |  |
| 0.39 | 200 (93.4) | 14 (6.5 %) | 0.51 | 206 (96.2%) | 8 (3.7%) | **Caregiver present (N %)** |
|  | 66 (90.4%) | 7 (9.5%) |  | 72 (98.6 %) | 1 (1.36%) | **Caregiver not present (N %)** |
|  | 266 (92.6 %) | 21 (7.3 %) |  | 278 (96.8%) | 9 (3.1%) | **Total (N %)** |

* Statistically significance was set at p<0.05; difference was calculated between Caregiver present and Caregiver not present.
